# Post-Market Non-Controlled Study on the Clinical Safety of a Synthetic Calcium Phosphate Ceramic in Alveolar Bone Regeneration: A 6-Month Prospective Study

**DOI:** 10.3390/jfb17050229

**Published:** 2026-05-06

**Authors:** Nuno Silva, Carlota Rodrigues, Angel Lobito, António Mano Azul, Pedro Ferreira Trancoso, Vanessa Machado, João Botelho

**Affiliations:** Egas Moniz Center for Interdisciplinary Research (CiiEM), Egas Moniz School of Health & Science, 2829-511 Almada, Portugal; nsilva@egasmoniz.edu.pt (N.S.); alobito@egasmoniz.edu.pt (A.L.); ptrancoso@egasmoniz.edu.pt (P.F.T.); vmachado@egasmoniz.edu.pt (V.M.)

**Keywords:** bioceramics, β-tricalcium phosphate, bone regeneration, oral surgery, prospective study

## Abstract

This prospective, single-arm post-market study aimed to evaluate the clinical safety and performance of a synthetic calcium phosphate ceramic used in alveolar bone regeneration procedures. Eighty adult patients requiring bone augmentation were treated with β-tricalcium phosphate (β-TCP) under routine clinical indications. Surgical approaches were adapted to defect morphology. Safety outcomes included adverse events (AEs) and device deficiencies (DDs), while performance outcomes focused on two-dimensional radiographic bone assessment. Radiographic bone consolidation was defined as continuous trabecular radiopacity without radiolucent defects or clinical signs of infection. Patients were followed for six months post-surgery, with clinical and radiographic evaluations, as well as assessment of oral health-related quality of life (OHIP-14). All 80 patients (mean age: 47.2 ± 18.9 years; 51% male) completed the immediate postoperative assessment. Eleven DDs (granule loss) were observed postoperatively (13.8%) and no AEs. At six months, 71 patients (88.8%) completed follow-up. Radiographic bone repair was confirmed in all cases clinically observed and with follow-up X-ray (100%). No AEs or DDs reported (AE-free rate: 100%) at this follow-up. The median OHIP-14 score improved significantly at six months (*p* = 0.037), indicating better self-reported oral health. Given the observational design, absence of a control group, and partial reliance on non-radiographic follow-up, these findings should be interpreted with caution. Within these limitations, the synthetic calcium phosphate ceramic demonstrated a favorable short-term safety profile and apparent bidimensional radiographic signs of clinical performance under real-world conditions, rather than definitive evidence of effectiveness. Further controlled studies incorporating histological and volumetric analyses are warranted to confirm its regenerative potential.

## 1. Introduction

Synthetic calcium phosphate (CaP) ceramics, including hydroxyapatite (HA), β-tricalcium phosphate (β-TCP), and biphasic calcium phosphate (BCP), have gained broad acceptance due to their biocompatibility, osteoconductive behavior, and structural similarity to the mineral phase of bone [1,2,3]. These materials provide a porous scaffold that supports angiogenesis and new bone formation and have demonstrated favorable clinical performance in diverse oral and maxillofacial applications, including alveolar ridge preservation, periapical defect regeneration following apicectomy, peri-implant defect management, and post-extraction socket healing [2,4,5].

Tooth extraction initiates a cascade of biological events that invariably result in dimensional alterations of the alveolar ridge [6]. Following removal of the tooth, disruption of the periodontal ligament eliminates the vascular supply to the bundle bone, leading to rapid osteoclastic activity and resorption of the socket walls. This process is particularly pronounced at the buccal plate, which is often thin and composed largely of bundle bone dependent on periodontal ligament perfusion. Consequently, collapse of the facial aspect of the ridge frequently occurs within the first weeks after extraction. Human clinical and radiographic studies have demonstrated that the greatest dimensional changes occur during the first three to six months of healing, with horizontal bone loss ranging between 29 and 63% and vertical reductions of approximately 11–22% [7]. These changes may continue, albeit at a slower rate, for up to two years.

Progressive post-extraction ridge contraction frequently reduces alveolar dimensions below those considered adequate for prosthetically guided implant placement, thereby increasing the need for additional augmentation procedures. Quantitative evidence indicates that, within the first six months after extraction, the alveolar ridge may undergo mean horizontal reductions of approximately 3.8 mm and vertical reductions of 1.24 mm, with the buccal plate being particularly susceptible to collapse [8]. In the aesthetic zone, dimensional changes may be even more pronounced. Farmer and Darby [9] reported that 42% of sites experienced ≥4 mm buccal bone loss within 6–8 weeks, and all implants subsequently required simultaneous augmentation. From a restorative standpoint, ridge width is critical for predictable implant positioning. Lee et al. [10] demonstrated that without ridge preservation, only 35% of patients achieved a preimplant ridge width ≥6 mm, compared with 82.5% when preservation procedures were performed. Nevertheless, even preservation strategies do not universally eliminate the need for further grafting, as simultaneous augmentation at implant placement may still be necessary in compromised sites [11]. Moreover, recent analyses suggest that a residual buccal thickness of approximately 2 mm is associated with reduced vertical resorption, underscoring that even partially preserved ridges may remain below biologically and prosthetically optimal dimensions [12]. Biomaterial selection is thus critical for optimizing both short- and long-term clinical outcomes.

Clinical outcomes of regenerative procedures are influenced not only by the biomaterial used but also by patient-related factors such as smoking habits, systemic conditions (e.g., diabetes and hypertension), and general health status, which may affect wound healing and bone metabolism [13,14]. Therefore, evaluating biomaterial performance under real-world clinical conditions, across heterogeneous patient populations and varying surgical indications, is essential. Alveolar bone regeneration is a cornerstone of contemporary oral rehabilitation and is essential for predictable outcomes in procedures such as dental implant placement, management of periapical pathology, and reconstruction following tooth extraction or explantation. Insufficient alveolar bone volume and quality may compromise both functional and aesthetic outcomes, making regenerative procedures a routine component of clinical practice [15,16]. Although autogenous bone grafting remains the clinical gold standard due to its osteogenic and osteoinductive properties, its use is limited by donor-site morbidity, increased surgical time, and restricted availability [17,18,19,20,21]. These limitations have encouraged the development and widespread use of alternative biomaterials. Allografts reduce surgical burden but may face lower patient acceptance and theoretical concerns regarding disease transmission. Xenografts, particularly bovine-derived hydroxyapatite, offer long-term space maintenance but display slow resorption kinetics, frequently resulting in residual particles that may delay complete replacement by vital bone. In contrast, fully synthetic alloplastic substitutes such as β-tricalcium phosphate provide standardized composition, unlimited availability, absence of immunogenicity, and predictable biodegradation [22]. These characteristics make synthetic calcium phosphates especially attractive for routine clinical use and large-scale implementation, motivating further evaluation of their real-world safety and performance.

Regulatory frameworks increasingly emphasize the importance of post-market clinical follow-up to confirm device safety and performance in routine practice. Prospective post-market studies offer valuable real-world evidence by capturing outcomes across everyday clinical scenarios, complementing data from preclinical and controlled clinical investigations [23,24,25]. Accordingly, the aim of this study was to evaluate the clinical safety and performance of a synthetic calcium phosphate ceramic used in routine alveolar bone regeneration procedures, including but not limited to alveolar ridge preservation, through a post-market prospective study with a six-month follow-up.

We hypothesize that this specific β-TCP formulation demonstrates a favorable safety profile and radiographic signs of two-dimensional bone regeneration in real-world clinical conditions, without increasing complication rates compared to expected standards of care.

## 2. Materials and Methods

### 2.1. Study Design and Setting

The present single-center, single-arm prospective cohort study, descriptive post-market clinical study is reported following the Transparent Reporting of Evaluations with Nonrandomized Designs (TREND) [26]. It was conducted in accordance with the ethical principles outlined in the Declaration of Helsinki, updated in 2024, and was approved by the Egas Moniz Ethical Committee (ID 1535/25). The study was carried out at the Egas Moniz Dental Clinic, a university-based clinical setting specialized in oral surgery and regenerative procedures. Subject recruitment followed a consecutive sampling approach between 5 March 2025 and 16 April 2025. External monitoring was made to ensure independent data validation.

### 2.2. Participants

Eligible participants were adult patients (≥18 years) requiring alveolar bone regeneration in preparation for future implant placement or prosthetic rehabilitation. Inclusion criteria comprised the following: males or females aged > 18 years old; undergoing reconstructive dental surgeries who meet predefined criteria in Instructions For Use (IFU) of TriOSS^®^ and are described as exclusion criteria; able to sign an informed consent form (for data collection); candidates for bone augmentation procedures in the sinus or alveolar ridge. Exclusion criteria were patients with inability to understand consent and the objectives of the study; signs of local or systemic acute/active or chronic infections; uncontrolled metabolic affections; severe degenerative diseases, conditions in which general bone grafting is not advisable; implementation sites that allow for product migration; conditions which require structural support in the skeletal system; conditions where the implantation site is unstable and not rigidly fixated; sensibility to the implantable materials; and known hypersensitivity to the implant material.

### 2.3. Intervention

In this study, we tested a fully synthetic bone graft material composed of β-TCP (TriOSS^®^, Bioceramed, Guimarães, Portugal). The device was assessed under routine clinical conditions for safety and performance. Immediate and six-month postoperative evaluations were conducted to monitor adverse events. Surgical duration was also recorded in minutes for all procedures.

All procedures were performed under local anesthesia using a standardized surgical protocol. The β-TCP used was in granules with a porosity > 40% (60–70%), macropores size > 500 µm, micropores size < 10 µm, uneven surface, compressive strength > 10.0 MPa and is radiopaque. The batch numbers of the medical devices used during the study were recorded in the case report forms. Two main batches were used: LOT 21T026457 (n = 55) and LOT 21T026478 (n = 24), plus one isolated entry (LOT 21T926457). Surgical procedures were performed by seven clinicians with clinical experience in oral surgery and implant-related procedures ranging from 2 to 30 years. All operators were routinely trained in the clinical use of synthetic bone graft materials as part of their clinical practice and followed the same standardized surgical protocol throughout the study to ensure procedural consistency.

Whenever feasible, minimally traumatic extraction techniques were employed using periotomes and luxators to preserve socket walls. Following extraction, sockets were thoroughly debrided and irrigated with sterile saline to remove granulation tissue. The β-TCP granules were hydrated with sterile saline prior to placement and gently compacted to fill the defect without excessive pressure, ensuring maintenance of intergranular porosity for vascular infiltration. Primary closure was achieved using interrupted 3-0 or 4-0 sutures. Postoperative care included systemic antibiotics when indicated, non-steroidal anti-inflammatory drugs for pain control, chlorhexidine rinses (0.12–0.2%) for 7–10 days, and standardized oral hygiene instructions.

The Portuguese-validated version of the Oral Health Impact Profile (OHIP-14) [27], which has been validated, was employed to assess how oral health affects the quality of life for each participant in the study. The OHIP-14 includes 14 questions designed to evaluate the degree to which oral health issues disrupt various aspects of a patient’s life, such as physical pain, physical disability, psychological discomfort, and social discomfort, among others [28]. This questionnaire reveals the frequency with which the individual encounters these situations, with responses rated on a 5-point scale for each question (never, hardly ever, occasionally, often, very often) [27,28].

Radiographic evaluation was standardized using periapical radiographs acquired with paralleling technique holders. Images were calibrated using proprietary software to allow for reproducible linear measurements of ridge height and width. Bone repair was defined as the presence of continuous trabecular radiopacity within the grafted site without radiolucent voids or clinical signs of infection. All radiographs were evaluated independently by one calibrated clinician blinded to clinical outcomes. Intra-observer reliability was evaluated using intraclass correlation coefficients (ICCs), yielding an excellent agreement (ICC = 0.96).

Radiographic bone repair was defined as continuous trabecular radiopacity without radiolucent defects or clinical signs of infection in 2D periapical X-ray [29].

Adverse events (AEs) and device deficiencies (DDs) were defined according to. An AE was defined as any untoward medical occurrence in a participant during the study period, regardless of whether it was considered related to the investigational device or study procedures. A DD was defined as any inadequacy of the investigational medical device related to its identity, quality, durability, reliability, safety, or performance, including malfunctions or inadequacies in the instructions for use, in accordance with ISO 14155:2020. Surgical complications were classified according to the Clavien-Dindo classification [30].

### 2.4. Variables and Data Sources/Measurement

The primary variables analyzed were the safety outcomes (e.g., adverse events, postoperative complications such as infection, or graft exposure) and clinical performance outcomes, assessed through radiographic and clinical evaluation of bone fill and graft integration over time. Baseline demographic and clinical variables—including age, sex, smoking status, systemic health status, and graft site characteristics—were recorded from patient charts and standardized clinical examination forms.

### 2.5. Sample Size

The sample size was informed by previously published clinical data on biphasic calcium phosphate granules [31]. Under a conservative paired design assumption (two-sided α = 0.05, power = 80%, within-subject correlation = 0.5), a minimum of 11 participants would be sufficient to detect this difference. However, as the primary objective of the present investigation was to evaluate clinical safety and real-world performance in a post-market setting across heterogeneous surgical indications, a pragmatic sample size was adopted. Consequently, a total of 80 patients was enrolled to enhance the external validity of the findings, allow for meaningful assessment of adverse events, and align with recommended sample sizes for post-market clinical follow-up studies of medical devices. This sample size was deemed sufficient to describe early clinical safety and performance trends in a real-world post-market context, in line with similar studies in the field.

### 2.6. Quantitative Variables

Quantitative data included continuous variables such as age (in years), time of intervention (in minutes) and visual analogue scale (0–10). Categorical variables such as sex, smoking status, and the presence/absence of postoperative complications were also assessed.

### 2.7. Statistical Analysis

Statistical analyses were conducted using R software (Version 4.0, R Foundation for Statistical Computing, Vienna, Austria). The distribution of continuous variables was assessed using the Shapiro–Wilk test. As several variables deviated from normal distribution (*p* < 0.05), non-parametric statistical methods were applied.

The primary endpoint was defined as the incidence of AEs within the 6-month follow-up period, while bidimensional radiographic bone consolidation and patient-reported outcomes were considered secondary endpoints.

Continuous variables were expressed as mean ± standard deviation for descriptive purposes, while categorical variables were presented as percentages. OHIP-14 domain and total scores were treated as continuous variables in the statistical analyses. The Mann–Whitney U test was used to compare continuous variables between two groups, and the Kruskal–Wallis test was applied when comparisons involved more than two groups. Fisher’s exact test was used to analyze categorical variables. These tests evaluate whether statistically significant differences exist in the distribution of variables between groups or categories. A significance level of *p* < 0.05 was adopted for all statistical analyses.

OHIP-14 scores were compared between participants who completed follow-up and those who did not using independent-samples *t*-tests with Welch correction, performed separately for each domain; statistical significance was set at *p* < 0.05, with adjustment for multiple comparisons where appropriate. Changes in OHRQoL were assessed using the Oral Health Impact Profile (OHIP-14). A radar (spider) plot was constructed to visually represent the mean change (Δ) in OHIP-14 domain scores between baseline (Visit 1) and the 6-month follow-up (Visit 2). The plot included the seven OHIP-14 domains: Functional Limitation, Physical Pain, Psychological Discomfort, Physical Disability, Psychological Disability, Social Disability, and Handicap. For each domain, mean delta values (Visit 2–Visit 1) and corresponding standard deviations were plotted on a circular coordinate system, with each axis representing one domain. The central point (0) indicated no change, and negative values denoted improvement in OHRQoL.

## 3. Results

### 3.1. Participants

A total of 93 individuals were invited to enroll in the study, 13 refused and with a final sample of 80 participants being included. Patients were sex-balanced (41 males and 39 females) with an average of 47.2 years old (±18.9, ranging between 19 and 82 years old). Nearly a quarter of participants were active smokers (n = 21, 26.3%). More than half were overweight (BMI ≥ 25) (n = 33, 41.3%) or obese (BMI ≥ 30) (n = 14, 17.5%), with an average of 26.3 years old (±4.3, ranging between 16.3 and 42.8 years old).

In terms of systemic health, 18 patients reported to suffer from hypertension (22.5) and 13 from diabetes mellitus (16.3%). There were no cases of osteopenia or osteoporosis.

The average operative time was 63.1 min (±25.0) ranging between 20 and 145 min. Only one case had peri-operative complications, with a fracture of tooth being removed, which required more surgical time. The majority of patients underwent alveolar ridge preservation, which was performed in 76 cases (95%). Other surgical procedures were considerably less common, each observed in only one patient (1.25%). These included alveolar ridge preservation with immediate removable prosthesis, apical filling after apicectomy, explantation followed by alveolar ridge preservation, and post-extraction with immediate implantation and provisionalization with a surgical hood.

**Figure 1:**
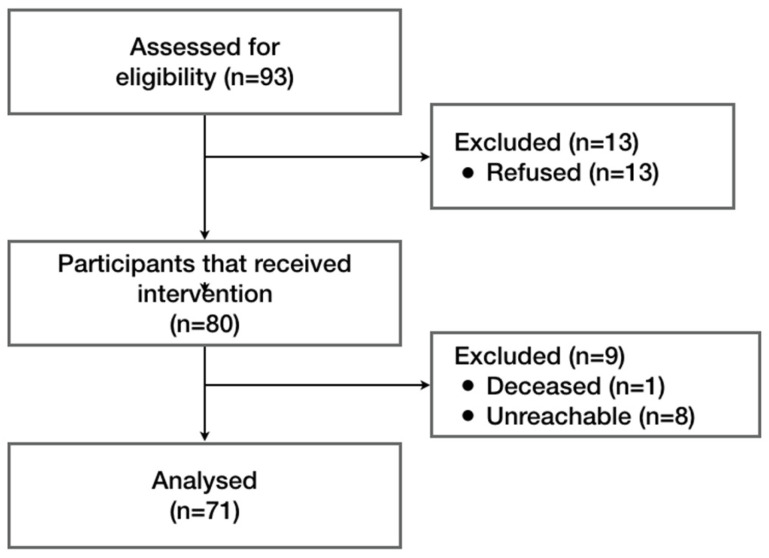
Flow diagram of participants. A total of 93 individuals were assessed for eligibility, of whom 13 declined to be enrolled. Eighty participants were enrolled and received the intervention (alveolar bone regeneration with TriOSS^®^). At the 6-month follow-up, nine participants were excluded, resulting in 71 patients included in the final analysis.

At the 6-month follow-up, 71 patients participated (a rate of 88.8%) (Figure 1). One patient had passed away from unrelated reasons to the trial purpose, and 8 were unreachable. The average age of the lost patients was 44.9 (±20.0) with 4 females and 5 males. In the included participants, average age was 47.2 (±18.8) with 37 males and 34 females. We found no differences between included and lost patients.

Tooth location was systematically recorded for all cases in the variable “*defect location.*” The treated sites included both anterior and posterior teeth, although the majority corresponded to posterior regions. The most frequently treated sites were third molars, including tooth 48 (n = 10), 18 (n = 8), 38 (n = 4) and 28 (n = 4). Other commonly treated sites included first and second molars, such as 26 (n = 6), 46 (n = 6), 36 (n = 4) and 37 (n = 4). A smaller number of cases involved premolars and anterior teeth.

### 3.2. Safety

Safety outcomes were prospectively evaluated at two predefined time points: the immediate postoperative period and the 6-month follow-up.

During the early postoperative phase, no AEs were observed among the 80 treated patients. A total of 11 DDs were observed corresponding to an incidence of 13.8% (95% CI: 7.1–23.3%), and consisted exclusively of minor granule loss from the grafted site. These occurrences were detected during early wound healing and were attributed to superficial particle displacement or mechanical migration rather than biological intolerance or device malfunction. No infections, allergic reactions, persistent inflammation, wound dehiscence, or graft rejection were recorded. All AEs were classified as Grade 1 in severity, were self-limiting, and did not require pharmacological or surgical intervention. Healing progressed uneventfully in all affected cases, and no treatments were discontinued.

No device deficiencies related to manufacturing defects, structural integrity, or handling characteristics were identified intraoperatively or postoperatively.

At the 6-month follow-up, 71 patients (88.8%) completed clinical and radiographic evaluation. Importantly, no new or ongoing adverse events or device deficiencies were reported at this time point, resulting in a late AE-free rate of 100% among evaluated participants. This indicates complete resolution of the transient early events and absence of delayed or chronic complications. Clinical examination confirmed normal soft tissue healing, absence of infection or foreign body reactions, and stable graft integration in all cases. Safety outcomes were comparable across subgroups, including smokers and patients with systemic conditions such as hypertension or diabetes mellitus.

### 3.3. Performance

**Figure 2:**
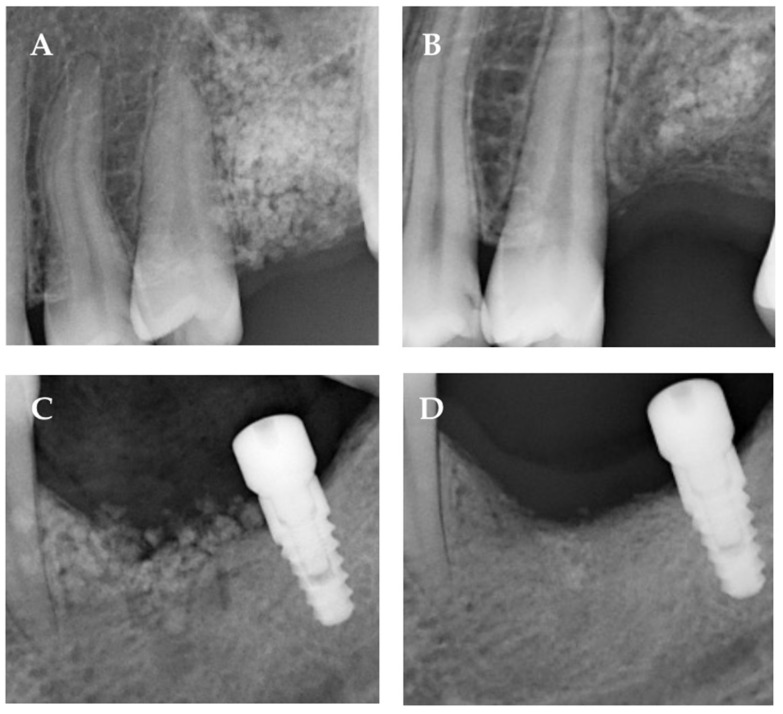
**Radiographic evolution of bone regeneration in two representative clinical cases.** (**A**,**B**) Case 1: Periapical radiographs obtained immediately after graft placement (**A**) and at the 6-month follow-up (**B**). The images show progressive trabecular pattern development with increased radiopacity within the grafted area, suggestive of ongoing bone formation and graft integration. (**C**,**D**) Case 2: Periapical radiographs acquired at the time of implant placement (**C**) and after 6 months of functional healing (**D**). The images demonstrate maintenance of marginal increasing levels and increased radiographic density in the peri-implant region, consistent with bone maturation and stable osseointegration. These images are presented for illustrative purposes only and do not constitute a standardized quantitative or comparative radiographic assessment. Due to the inherent limitations of two-dimensional radiography, no linear or volumetric measurements were performed.

Bone consolidation was radiographically and clinically confirmed in all 71 patients who attended the 6-month follow-up (100%), demonstrating consistent and apparent bone regeneration at the grafted sites (Figure 2). Radiographic evaluation revealed progressive trabecular bone formation, increased radiopacity within the defect area, and maintenance of ridge contour, findings compatible with successful graft integration and ongoing remodeling. No radiolucent defects, inflammatory signs, or graft-related complications were detected.

In six cases where standardized radiographic records were unavailable at the follow-up visit, bone healing was assessed through structured teleconsultation using a physician-administered clinical questionnaire. This assessment focused on the absence of local symptoms such as pain, swelling, suppuration, soft-tissue dehiscence, or delayed healing. All six patients reported uneventful recovery and absence of clinical complications, supporting satisfactory healing in these cases. These assessments were used to complement, rather than replace, radiographic evaluation and were considered acceptable within the context of this real-world post-market study.

With regard to functional performance, 65 patients (91.6%) were classified as presenting bone regeneration adequate for dental implant placement based on clinical and radiographic criteria, including ridge width, height, and bone density compatible with primary implant stability. The remaining six patients (8.45%) were categorized as “inconclusive,” primarily due to limited imaging availability or the need for additional healing time rather than confirmed regenerative failure. Importantly, no sites were classified as inadequate or unsuccessful.

### 3.4. Oral Health-Related Quality of Life

We started by comparing baseline OHIP-14 overall and domain scores between participants who completed follow-up and those who did not (Table 1). A significant difference was observed for functional limitation (*p* = 0.0049), while no differences were found for the remaining domains or total score (all *p* > 0.05), except for handicap (*p* = 0.017), which did not remain significant after multiple comparison correction.

**Figure 3:**
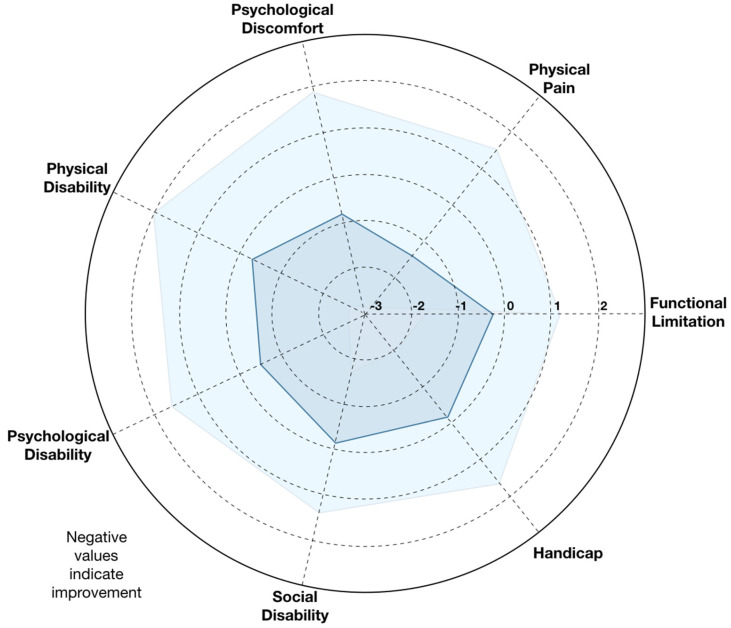
Radar plot of OHIP-14 domain score changes from baseline to 6 months. Radar plot illustrating the mean change (Δ) in Oral Health Impact Profile (OHIP-14) domain scores between baseline and six months post-surgery. Each axis represents one of the seven OHIP-14 domains: Functional Limitation, Physical Pain, Psychological Discomfort, Physical Disability, Psychological Disability, Social Disability, and Handicap. Negative values indicate improvement in oral health-related quality of life (OHRQoL). Overall, reductions were observed across all domains, with the most notable improvements in Physical Pain and Psychological Discomfort, followed by Physical Disability. Smaller yet consistent improvements were evident in the remaining domains, reflecting a general enhancement in self-perceived oral health following treatment with the synthetic calcium phosphate ceramic (TriOSS^®^). Light blue represents the 95% CI, dark blue represents the average score.

At the 6-month follow-up (n = 71), the mean OHIP-14 score significantly decreased to 5.1 ± 9.5, corresponding to an average impact of 9.2% (±16.9%), reflecting an overall improvement in oral health-related quality of life (OHRQoL) (Figure 3). Sixteen participants maintained a score of 0 at both time points, while an additional 24 patients achieved complete resolution of symptoms, reaching an OHIP score of 0 after treatment. These findings indicate that nearly one-third of the cohort experienced full restoration of perceived oral well-being following regenerative therapy.

Among participants who completed follow-up, significant reductions were observed in OHIP-14 total score (*p* = 0.018), physical pain (*p* < 0.001), psychological discomfort (*p* = 0.013), and psychological disability (*p* = 0.040); however, after Bonferroni correction for multiple comparisons, only the physical pain domain remained statistically significant (adjusted *p* = 0.001) (Table 1).

Domain-level analysis revealed consistent reductions across all seven OHIP-14 dimensions. The largest improvements were observed in Physical Pain and Psychological Discomfort, followed by Physical Disability, reflecting decreased postoperative discomfort and improved masticatory function. Smaller but uniform improvements were recorded for Functional Limitation, Social Disability, and Handicap domains, suggesting broader psychosocial benefits beyond purely clinical healing. The radar plot (Figure 2) illustrates these multidimensional changes, with negative delta values across all axes indicating improvement in every domain.

Fourteen participants reported slightly higher OHIP scores at six months; however, none of these complaints were attributable to the implanted biomaterial or surgical site and were instead associated with unrelated dental or systemic issues. Importantly, no patient reported persistent pain, graft-related discomfort, or functional limitations directly linked to the intervention.

## 4. Discussion

The present post-market prospective study evaluated the clinical safety and performance of a synthetic calcium phosphate ceramic in several surgical procedures, mainly alveolar bone regeneration procedures. Over a 6-month follow-up, no serious AEs were reported, and all treated sites in the present cohort demonstrated radiographic bone consolidation. These findings suggest an acceptable safety profile and consistent clinical performance within the observational conditions of this study, although definitive conclusions remain limited by the study design.

Our results are consistent with previous evidence supporting the excellent biocompatibility and osteoconductivity of synthetic calcium phosphate ceramics, including hydroxyapatite (HA), β-tricalcium phosphate (β-TCP), and biphasic calcium phosphate (BCP). These materials have long been recognized for their structural and chemical similarity to native bone mineral, providing a scaffold that supports new bone formation while gradually resorbing in vivo. The absence of adverse events or postoperative complications at 6 months in our cohort further corroborates the biological inertness of these materials is in line with prior reports, which describe minimal inflammatory response and integration in both animal and human studies [22,32,33].

β-tricalcium phosphate promotes bone regeneration primarily through osteoconduction and controlled biodegradation [34]. Following implantation, partial dissolution of β-TCP releases calcium and phosphate ions into the surrounding microenvironment, stimulating osteoblastic differentiation and mineral deposition [35]. Simultaneously, its interconnected porous structure facilitates angiogenesis and migration of osteoprogenitor cells [36]. As the material gradually resorbs, it is replaced by newly formed vital bone through a process of creeping substitution [37]. This balance between degradation and formation is thought to support ridge preservation, although variability between patients and defect types should be considered.

The present findings align with prior clinical studies reporting high rates of bone consolidation and implant-ready bone formation following the use of synthetic calcium phosphate grafts in ridge preservation and augmentation procedures [38]. For example, pure-phase β-TCP grafting has been shown to preserve socket dimensions and support subsequent implant placement with regenerated sites demonstrating adequate density and stability at reentry around 4–6 months post-grafting [39]. Previous systematic evaluations suggest that synthetic calcium phosphate biomaterials may achieve outcomes comparable to natural grafts [40]. Within this context, the present results and absence of serious adverse events fall within or above reported clinical ranges, reinforcing the reliability of synthetic β-TCP under heterogeneous routine clinical conditions. Moreover, improvements in patient-reported quality of life in our cohort are consistent with broader evidence that regenerative procedures using minimally invasive alloplastic materials can enhance functional recovery and satisfaction by reducing discomfort and disability associated with alveolar defects [40].

An additional strength of this study lies in the post-market surveillance design under a real-world design, which provides real-world evidence on product safety and performance across a heterogeneous patient population. Unlike controlled clinical trials that often include selected patients, this pragmatic approach enhances external validity by reflecting actual clinical practice, including variable comorbidities, surgical techniques, and defect morphologies. The inclusion of smokers (26.3%), hypertensive and diabetic individuals, conditions often associated with delayed bone healing, adds robustness to the results, as no adverse AEs were reported at 6 months even in these subgroups.

The observed improvement in oral health-related quality of life (OHRQoL) after surgery, reflected by a decrease in OHIP-14 scores from 8.2 to 5.1, further supports the clinical benefit of this biomaterial. However, these findings should be interpreted cautiously, as bone substitutes primarily aim to restore structural integrity, their impact on patient well-being and functional recovery is increasingly recognized as an essential endpoint in regenerative procedures using synthetic biomaterials.

Despite its strengths, this study presents some limitations. The sample size, though adequate for post-market surveillance, limits the statistical power to detect rare complications or subgroup differences. The absence of a control group is a central limitation and precludes direct comparison with other grafting materials and prevents any conclusions regarding superiority or equivalence. Radiographic bone gain was not quantified volumetrically which restricts precision in assessing bone density evolution. Another limitation of this study is that dimensional changes in the alveolar ridge were not systematically recorded during follow-up, which limits the ability to quantify volumetric or linear alterations occurring after socket grafting. Moreover, the 6-month follow-up provides early insight into bone healing but does not capture long-term resorption or remodeling behavior, which may differ among patients and biomaterials. The loss to follow-up was also observed and adds some shortcoming to the results analysis. Future studies with extended follow-up periods, histological validation, and comparison to alternative grafting materials are warranted to comprehensively assess the long-term performance of this material. Regarding buccal bone phenotype, this parameter was not systematically recorded in the study protocol and should be accounted for in future studies. Another limitation relates to the remote administration of patient-reported outcome measures. OHIP-14 was collected through teleconsultation, which may introduce response variability compared with in-person administration. However, telephone-based OHIP collection has been previously validated and used in clinical research [41].

Future investigations should incorporate longer follow-up periods exceeding 24 months to assess long-term dimensional stability and implant survival. Volumetric CBCT analyses and digital intra-oral scanning could provide more precise three-dimensional quantification of ridge preservation. Histological or histomorphometric assessments would further elucidate the proportion of vital bone versus residual biomaterial. Randomized controlled trials comparing β-TCP with xenografts or autogenous bone would clarify relative effectiveness and cost-benefit profiles. Such studies would help determine the extent to which the present findings can be generalized to broader clinical settings.

## 5. Conclusions

This post-market observational study indicates that the tested synthetic calcium phosphate ceramic was associated with acceptable short-term safety and radiographic signs of bone regeneration, together with improvements in patient-reported outcomes up to six months after surgery. Given the non-comparative design and limited follow-up period, these findings should be interpreted with caution. Further controlled studies with longer observation times are needed to confirm its long-term safety and clinical effectiveness.

## Figures and Tables

**Table 1:** OHRQoL at baseline and 6 months of follow-up. Results are presented as mean ± SD.

OHIP-14, Mean (SD)	Baseline (n = 80)	Baseline (n = 71)	Baseline of Drop-Out Patients (n = 9)	*p*-Value (Completed vs. Drop-Out) *	Follow-Up (n = 71)	Baseline vs. Follow-Up (n = 71) **
Total	8.2 (10.9)	8.6 (11.3)	9.7 (9.6)	0.307	5.0 (9.5)	0.018
Functional limitation	0.7 (1.4)	0.7 (1.5)	0.2 (0.7)	0.001	0.4 (1.1)	0.137
Physical pain	2.4 (2.5)	2.4 (2.5)	2.0 (1.4)	0.905	1.0 (1.9)	<0.001
Psychological discomfort	1.6 (2.4)	1.7 (2.5)	2.4 (1.7)	0.602	0.9 (1.7)	0.013
Physical disability	1.2 (2.1)	1.2 (2.1)	1.6 (2.2)	0.242	0.9 (1.7)	0.266
Psychological disability	1.2 (1.8)	1.3 (1.9)	1.8 (2.2)	0.454	0.8 (1.6)	0.040
Social disability	0.5 (1.3)	0.6 (1.3)	0.8 (1.1)	0.207	0.5 (1.3)	0.401
Handicap	0.6 (1.4)	0.6 (1.5)	0.9 (1.8)	0.017	0.5 (1.4)	0.429

* Bonferroni-adjusted *p*-values (multiplication by eight comparisons) are reported for each domain (OHIP-14 total: 1.000; functional limitation: 0.039; physical pain: 1.000; psychological discomfort: 1.000; physical disability: 1.000; psychological disability: 1.000; social disability: 1.000; handicap: 0.134); only the functional limitation domain remained statistically significant after correction. ** Bonferroni-adjusted *p*-values (multiplication by eight comparisons) were calculated for each domain (OHIP-14 total: 0.142; functional limitation: 1.000; physical pain: 0.001; psychological discomfort: 0.103; physical disability: 1.000; psychological disability: 0.319; social disability: 1.000; handicap: 1.000); only the physical pain domain remained statistically significant after correction.

## Data Availability

The original contributions presented in the study are included in the article, further inquiries can be directed to the corresponding author.
